# Optical coherence tomography findings in patients with transfusion-dependent β-thalassemia

**DOI:** 10.1186/s12886-022-02490-z

**Published:** 2022-06-24

**Authors:** Sezaneh Haghpanah, Omid Reza Zekavat, Sanaz Safaei, Mohammad Ali Ashraf, Shirin Parand, Hossein Ashraf

**Affiliations:** 1 Hematology Research Center, Shiraz University of Medical Sciences, Shiraz, Iran; 2 Student Research Committee, Shiraz University of Medical Sciences, Shiraz, Iran; 3 Poostchi Ophthalmology Research Center, Shiraz University of Medical Sciences, Shiraz, Iran

**Keywords:** β-thalassemia, Macular thickness, Optical coherence tomography, Retinal nerve fiber layer thickness

## Abstract

**Background:**

Structural ophthalmologic findings have been reported in patients with β-thalassemia due to chronic anemia, iron overload, and iron chelation therapy toxicity in few previous studies. We aimed to investigate structural ocular findings and their relationship with hematological parameters in patients with transfusion-dependent β-thalassemia (TDT).

**Methods:**

In this cross-sectional study, from January 2018 to January 2019, 39 patients with TDT over the age of 18 participated. Multicolor fundus imaging, optical coherence tomography (OCT), and blue light fundus autofluorescence imaging were performed for all patients and 27 age- and sex-matched controls.

**Results:**

The mean age of patients was 28.6 ± 6.2 years. The central macular thickness and macular thicknesses in all quadrants were significantly thinner in patients than controls (*P*<0.05). None of the retinal nerve fiber layer (RNFL) measurements were significantly different between TDT patients and controls. There was a significantly negative correlation between hemoglobin with central macula thickness (*r*=-0.439, *P*=0.005). All measurements of macular subfield thickness were insignificantly thinner in patients with diabetes mellitus (DM) compared to the non-DM subgroup.

**Conclusions:**

Macular thickness was significantly thinner in central macula and entire quadrants in TDT patients compared to healthy individuals; however, all RNFL measurement thicknesses were comparable between the two groups. Close monitoring of TDT patients by periodic ophthalmologic examinations with more focus on diabetic patients, patients with severe anemia and iron overload should be warranted.

## Background

Thalassemia is the most common hereditary blood disorder worldwide [1]. In β-thalassemia, genetic mutations mainly consist of two types including: complete absence (β0 thalassemia) or decreased synthesis (β+ thalassemia) of the β chains leading to ineffective erythropoiesis and hemolysis [2]. In the severe form of the disease, transfusion-dependent β-thalassemia (TDT), patients need lifelong blood transfusion, which in turn causes iron overload and multi-organ dysfunction. Iron chelation therapy (ICT) prevents the toxic effects of iron but has some uninvited adverse events. A wide range of abnormal clinical ophthalmologic findings has been previously reported in thalassemia patients [1, 3–5]. On the other hand, few studies have evaluated and focused on structural ocular changes such as retinal nerve fiber layer (RNFL) and macular thickness in these patients [6–8]. Optical coherence tomography (OCT) is a non-invasive and well-tolerated procedure with a high level of sensitivity to determine RNFL, macular thickness, and optic nerve head morphology, providing important clinical information and differential diagnosis of the optic nerve and macular diseases [9].

In this study, we evaluated structural ophthalmologic findings using multimodal imaging including OCT in a group of patients with TDT. Moreover, we investigated the association of these findings with clinical and hematological factors.

## Methods

In this cross-sectional study, from January 2018 to January 2019, 39 patients with TDT over the age of 18 participated. All patients were registered at the Thalassemia Comprehensive Center, affiliated with Shiraz University of Medical Sciences in Shiraz, Southern Iran. Diagnosis of TDT patients was based on clinical history and hemoglobin electrophoresis. Inclusion criteria were TDT patients with regular blood transfusion every 2-4 weeks to sustain hemoglobin levels between 9-11 g/dl and receiving one type of ICT regimen within the last two years. Iron chelation regimens consisted of deferoxamine, deferasirox, or deferiprone with appropriate dosage either as monotherapy or combination therapy. Exclusion criteria were coexistence of other hemoglobinopathies or other types of anemias, those with any congenital ocular abnormality, history of ocular trauma and surgery, glaucoma, high refractive errors (spherical equivalent > 6.00 diopters), retinal disorders including papilledema, papillitis, retinal vascular disorders, and retinal detachment and having no willingness to participate in the study. Recently, we presented clinical ocular findings in 79 TDT patients [10], accordingly, the patients who cooperated for more investigations by OCT have been discussed in the present study. Twenty-seven age-and sex-matched healthy individuals were selected as controls.

The study protocol was approved by the Ethics Committee of Shiraz University of Medical Sciences (grant number=11886). All procedures were performed in accordance with relevant guidelines. Informed consent was obtained from all patients.

A complete ophthalmic examination was done for all patients by an expert ophthalmologist. Snellen VA assessment and intraocular pressure (IOP) measurements were conducted for all patients and controls [10]. Multicolor fundus imaging, OCT of peripapillary RNFL and macular thickness map, and blue light autofluorescence (BAF) imaging were performed for all patients and controls as well. Quantitative and qualitative OCT findings were isolated and compared with the same parameters in 27 age-and sex-matched healthy individuals.

The BAF and OCT images and all of the measurements were performed by an experienced technician using Heidelberg HRA2 retinal angiography and Spectralis (Spectral Domain Optical Coherence Tomography, SD-OCT) instruments (Heidelberg Engineering, Dossenheim, Germany) respectively. The peripapillary RNFL thickness was evaluated globally and in four sectors consisted of superior, inferior, temporal, and nasal sectors. Only images ⩾25 dB were indicated by the blue quality bar accepted for analysis. In the statistical analysis of RNFL and macular thickness measurements, the values of the right eyes were considered. Global (average), superior, inferior, temporal, and nasal RNFL thicknesses, global RNFL asymmetry between the two eyes, macular thickness map and superior-inferior hemisphere asymmetry (S-I asymmetry) as well as macula central and superior, inferior, temporal, and nasal perimacular sub-field thicknesses using the inner ring of superimposed 6 mm Early Treatment Diabetic Retinopathy Study (ETDRS) map centered over the center of the fovea [11] were determined in the OCT image and compared between patients and controls. Multicolor fundus imaging was obtained using multicolor module of the Spectralis SD-OCT instrument (Heidelberg Engineering, Dossenheim, Germany).

Clinical and laboratory data were collected from the patients´ medical records including serum ferritin and pre-transfusion hemoglobin levels, splenectomy status, type of ICT regimen in the last two years, and the presence of diabetes mellitus (DM).

### Statistical analysis

Data analysis was performed by Statistical Package for the Social Sciences (SPSS Inc., Chicago, Illinois, USA) version 21. The normality of the data was checked by Kolmogorov-Smirnov test. In descriptive analysis, the data were summarized as mean and standard deviation (SD) or median and range in the absence of normal distribution as well as frequency and percentage. Inferential statistics used in this study included Student t-test and Mann-Whitney test to compare quantitative variables between two independent groups. Also, the correlation between two quantitative variables was calculated as Pearson correlation coefficient or Spearman correlation coefficient. Values of P less than 0.05 was considered statistically significant.

### Power analysis

Power analysis was done by R software version 3.6.1. For comparing retinal measurements between thalassemia patients and healthy individuals (main objective of the study) the calculated power was about 90%.

For assessing the correlation of hematologic parameters with retinal measurements in the thalassemia group (sub-objectives of the study), it was calculated as 81% in case of correlation of hemoglobin with central macular thickness (CMT). In other significant correlations, power ranged from 52% to 57%.

## Results

The mean age of patients and healthy individuals were comparable (28.6 ± 6.2 vs. 27.1 ± 6.0, respectively, *P*=0.356). Twenty-seven (69.2%) of the patients and 14 (51.9%) of healthy individuals were female (*P*=0.199). The mean ± SD of hemoglobin and serum ferritin levels were 9.8 ± 0.68 g/dl and 2694 ± 1874 ng/ml, respectively, in TDT patients. Twelve patients (30.8%) were splenectomized. Types of ICT regimens used in TDT patients consisted of combined deferoxamine (DFO) and deferiprone (DFP) (16, 41%), deferasirox (DFX) (12, 30.8%), combined DFO and DFX (6, 15.4%) and DFO (5, 12.8%). Thirteen patients (33.3%) had diabetes mellitus with a mean duration of 13 ± 5 (3-20) years.

The mean ± SD of the best-corrected visual acuity (VA) in patients was 0.941 ± 0.115 decimal, (range: 0.6-1), 0.029 ± 0.062 logMar, (range: 0-0.2). The frequency of patients with logMar VA >0.1 logMar was 3 (7.7%). The mean ± SD of IOP was 15.51 ± 3.25 (6-24) mmHg in TDT patients. Only one patient had IOP above 22 mmHg (24 mmHg).

In healthy subjects, the mean ± SD of the best-corrected VA was 0.904 ± 0.136 decimal, and it was 15.33 ± 2.60 mmHg for IOP. Both VA and IOP were comparable in patients and controls (*P*=0.271 and *P*=0.813, respectively).

Moreover, the median spherical equivalent refractive error in patients and healthy individuals were comparable (mean ±SD: -1.36 ± 1.37, median: -1, range: -5.75-0.9 diopters in patients; mean ±SD: -1.02 ± 1.42, median: -0.75, range: -4.75-1.2 diopters in controls, *P*=0.304).

Posterior segment examinations (both eyes) revealed fundus abnormalities in three patients consisting of one large cup-to-disk (CD) ratio (more than 30%), one vessel tortuosity, and one retinal pigment epithelium (RPE) mottling.

None of the RNFL measurements, global RNFL thickness asymmetry, macular thickness map, and S-I asymmetry in the macular thickness measurements were significantly different between TDT patients and controls (*P*>0.05) (Table 1). This comparison was conducted again after excluding DM patients, and the results were the same. The results of overall RNFL assessments were compared between patients and healthy individuals, which showed a non-significant higher frequency of abnormal/borderline cases in patients compared to controls in both eyes: OD: 6 (15.4%) in patients versus 3 (11.1%) in controls, (*P*=0.727), OS: 7 (17.9%) in patients versus 2 (7.4%) in controls (*P*=0.290).

**Table 1:** Comparison of the retinal nerve fiber layer, global asymmetry, macular thickness map, and S-I hemisphere asymmetry between transfusion-dependent β-thalassemia patients and controls

Groups Variables (μm)	Patients (*n*=39)	Controls (*n*=27)	*P*-value
	Mean ± SD	Mean ± SD	
**Global (Average) RNFL**	101.87 ±11.02	99.22 ±9.95	0.322
**Superior RNFL**	122.38 ± 24.01	120.51±14.56	0.720
**Inferior RNFL**	137.84 ± 20.94	131.29 ± 18.56	0.196
**Temporal RNFL**	72.35 ± 12.64	70.88 ± 9.20	0.607
**Nasal RNFL**	74.94 ± 15.55	74.11 ± 12.59	0.817
**Global RNFL asymmetry**	-0.487 ± 4.91 Median, range : 0, -18 -9	-1.07 ± 5.62 Median, range: -1, -14 -15	0.420
**Macular thickness map**	293.59 ± 13.55	293.68 ± 11.31	0.980
**S-I hemisphere asymmetry**^**a**^	6.10 ± 13.07 Median, range: 3, 0-68	3.75 ± 3.29 Median, range: 2, 0-9	0.483

The Student t-test was used for all comparisons between the two groups, except for global RNFL asymmetry and S-I asymmetry which Mann-Whitney test was used

*SD* Standard deviation, *RNFL* Retinal nerve fiber layer

^a^The absolute difference of average macular thickness between superior and inferior hemisphere

As shown in Table 2, the CMT, as well as macular thickness measurements in all quadrants, were significantly thinner in patients than the same measurements in controls (*P*<0.05) (Table 2). We repeated this analysis after excluding DM patients, and the results were equal except for CMT that was insignificantly thinner in TDT patients compared to healthy individuals.

**Table 2:** Comparison of the macular thickness measurements between transfusion-dependent β-thalassemia patients and controls

Groups Macular thickness (μm)	Patients *N*=39	Controls *N*=27	*P*-value
	mean ± SD	mean ± SD	
**Macula center**	248.59 ±22.80	260 ± 21.48	0.040
**Superior primacula**	329.64 ± 24.57	346.88 ± 15.49	0.002
**Inferior primacula**	327.33 ± 24.31	343.14 ± 15.18	0.004
**Temporal primacula**	314.89 ± 20.01	331.18 ± 16.99	0.001
**Nasal primacula**	328.38 ± 23.13	345.37 ± 17.10	0.002

Student t-test was used for all comparisons between the two groups

*SD* Standard deviation

**Fig. 1:**
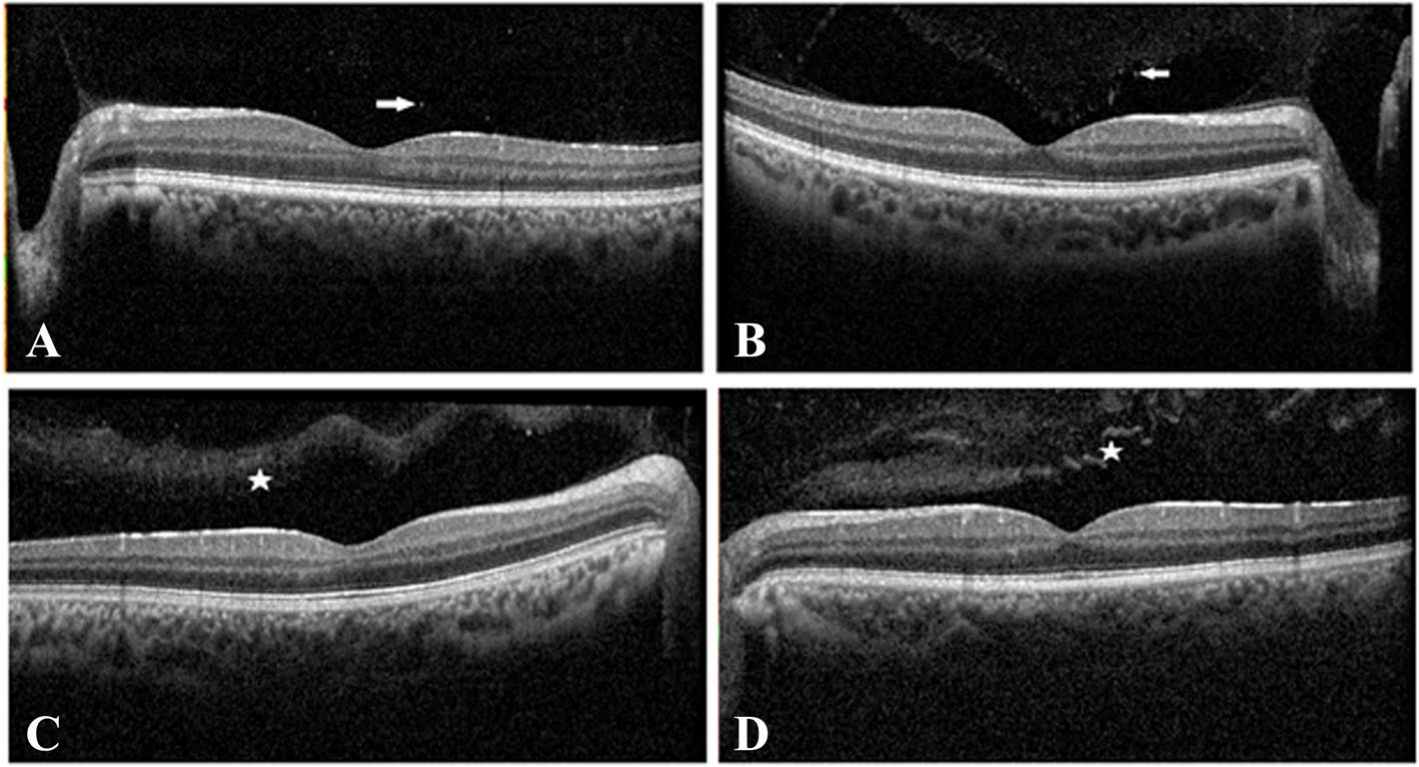
Optical coherence tomography of patients with transfusion-dependent β-thalassemia. A and B show preretinal vitreous cells as hyper reflective dots (white arrow); C and D show preretinal vitreous condensations (star)

Distribution of the frequency of abnormal findings in fundus images, OCT, and BAF of both eyes were summarized in Table 3. The most frequent findings included tigeroid patches: 13 (16.6%), RPE granularity: 10 (12.8%), foveolar or peripheral darkening: 8 (10.2%), hypo-autofluorescence patch: 6 (7.7%) and preretinal vitreous cell and condensations: 6 (7.7%) (Fig. 1).

**Table 3:** Frequency of abnormal findings in BAF, OCT and multicolor fundus image in 39 patients (both eyes) with transfusion-dependent β-thalassemia

Findings	N (%)
**Tigroid patches**	13 (16.6)
**RPE granularity**^**a**^	10 (12.8)
**Foveolar or peripheral darkening**	8 (10.2)
**Preretinal vitreous cell and condensations**	6 (7.7)
**Hypoautofluorescence patch**	6 (7.7)
**Hyper-reflective spots**	4 (5.1)
**Myopic fundus**	2 (2.5)
**Thin choroid**	2 (2.5)
**Cotton wool spots and hemorrhage**	1 (1.3)
**Disk drusen**	1 (1.3)
**Irregular radiating Hyperautofluorescence**	1 (1.3)
**Target like foveal BAF**	1 (1.3)

All percentages were calculated by eyes

^a^Retinal Pigment Epithelium granularity

**Fig. 2:**
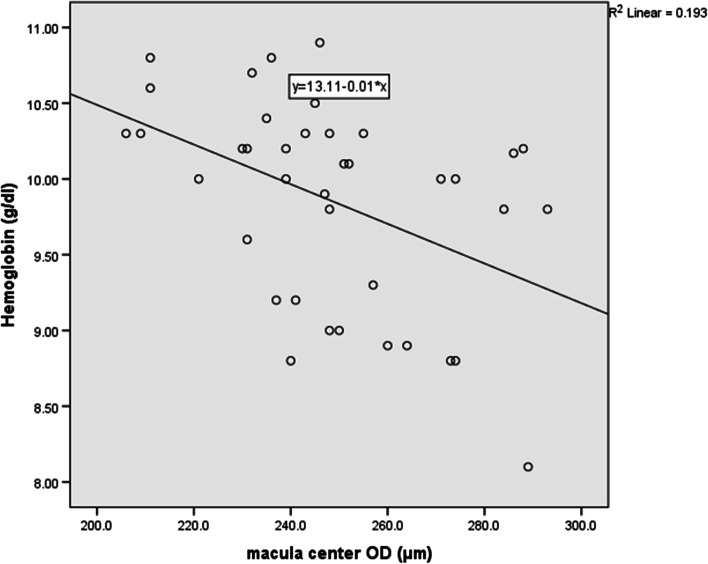
Correlation of hemoglobin level with macula center (r=-0.439, P=0.005)

Table 4 presents the correlation of macular thickness measurements with hemoglobin and serum ferritin levels and age. There were significant negative correlations between hemoglobin with central macula (*r*=-0.439, *P*=0.005, power: 81%) (Fig. 2) as well as nasal and temporal macula, but with low power (57% and 52% respectively) .The RNFL, global RNFL asymmetry, macular thickness map, and the S-I asymmetry in macular thickness measurements showed no statistically significant correlation with hemoglobin and, serum ferritin levels, and age, except for superior RNFL that showed a significant negative correlation with ferritin, but with low power (54%) (Table 5).

**Table 4:** Correlation of the macular thickness measurements with hemoglobin, and serum ferritin levels, age, and type of iron chelation therapy of the patients with TDT patients

(μm)	Hemoglobin (g/dl)		Ferritin (ng/ml)		Age (year)		Iron Chelation Therapy		
	Correlation coefficient	*P*-value	Correlation coefficient	*P*-value	Correlation coefficient	*P*-value	Mono therapy *n*=17 mean±SD	Combined therapy *n*=22 mean±SD	*P* value
**Macula center**	-0.439	0.005	-0.224	0.170	-0.092	0.577	251±25	246±20	0.486
**Superior primacula**	-0.300	0.064	-0.191	0.245	-0.019	0.909	331±23	328±25	0.727
**Inferior primacula**	-0.266	0.102	-0.219	0.181	-0.141	0.391	326±24	327±24	0.859
**Temporal primacula**	-0.319	0.048	-0.229	0.161	-0.016	0.925	314±21	314±19	0.984
**Nasal primacula**	-0.337	0.036	-0.265	0.103	-0.070	0.671	328±22	328±23	0.984

Pearson correlation coefficient was calculated in all measurements

For type of iron chelation therapy Student t-test was done

**Table 5:** Correlation of the retinal nerve fiber layer, global asymmetry, macular thickness map, and S-I hemisphere asymmetry measurements with hemoglobin and serum ferritin levels, age, and type of iron chelation therapy of the patients with transfusion-dependent β-thalassemia patients

(μm)	Hemoglobin (g/dl)		Ferritin (ng/ml)		Age (year)		Iron Chelation Therapy		
	Correlation coefficient	*P*-value	Correlation coefficient	*P*-value	Correlation coefficient	*P*-value	Mono therapy *n*=17 mean±SD	Combined therapy *n*=22 mean±SD	*P* value
**Global (average) RNFL**	-0.096	0.563	-0.267	0.100	-0.082	0.621	104±9	100±12	0.269
**Superior RNFL**	-0.107	0.516	-0.328	0.042	-0.090	0.584	125±16	120±28	0.539
**Inferior RNFL**	0.068	0.679	-0.192	0.241	0.048	0.774	141±16	135±24	0.407
**Temporal RNFL**	-0.235	0.150	0	>0.999	0.015	0.927	74±15	70±10	0.327
**Nasal RNFL**	-0.024	0.886	-0.005	0.974	-0.195	0.233	75±16	74±15	0.793
**Global RNFL asymmetry**	-0.084	0.610	-0.163	0.320	0.008	0.962	2(-10-9) median range	-0.5 (-18-5) median range	0.566
**Macular thickness map**	-0.182	0.267	-0.254	0.118	-0.218	0.183	293±11	293±15	0.851
**S-I hemisphere aymmetry**^**a**^	0.165	0.315	-0.064	0.697	0.262	0.107	3 (0-10) median range	2.5 (0-68) median range	0.624

Pearson correlation coefficient was calculated in all measurements except for global RNFL asymmetry and hemisphere S-I which Spearman correlation coefficients were measured. For type of iron chelation therapy Student t-test and Mann-Whitney test were done

^a^ The absolute difference of average macular thickness between superior and inferior hemisphere

The mean of RNFL measurements, global RNFL asymmetry, macular thickness map, and the S-I asymmetry in macular thickness measurements, as well as the macular thickness sub-field measurements, were compared amongst four groups of ICT regimens that showed no statistically significant differences (*P*>0.05, data not shown). Also, not any significant association was found with regard to ICT categorized into two groups of monotherapy or combination therapy (Tables 4 and 5). None of the aforementioned variables showed significant relationships with splenectomy, as well (*P*>0.05).

Table 6 shows the comparison of the mean ± SD of RNFL thicknesses, global RNFL asymmetry, macular thickness map, the S-I asymmetry in macular thickness measurements, and all macular subfields thickness measurements between TDT patients with and without DM. The differences were not statistically significant in either of the values. All measurements of macular subfield thickness were insignificantly thinner in patients with DM compared to the non-DM subgroup. Macular thickness was not correlated with the duration of DM in each of the evaluated measurements (*P*>0.05).

**Table 6:** Comparison of the retinal nerve fiber layer, global asymmetry, macular thickness map, S-I hemisphere asymmetry measurements, and macular thickness measurements between transfusion-dependent β-thalassemia patients with and without diabetes mellitus

Groups Variables (μm)	DM^a^ (*n*=13)	No DM (*n*=26)	*P*-value
	Mean ± SD	Mean ± SD	
**Average RNFL**	101.69 ± 15.76	101.96 ± 8.09	0.944
**Superior RNFL**	125.76 ± 24.17	120.69 ± 24.23	0.541
**Inferior RNFL**	136.92 ± 28.75	138.30 ± 16.40	0.849
**Temporal RNFL**	73 ± 13.99	72.03 ± 12.19	0.826
**Nasal RNFL**	71.07 ± 17.29	76.88 ± 14.58	0.277
**Global asymmetry**	0.692 ± 4.80 Median, range: 2 (-7-6)	-1.077 ± 4.95 Median, range:-0.5 (-18-9)	0.295
**Macular thickness map**	293.46 ± 16.40	293.65 ± 13.89	0.967
**S-I Asymmetry**^**b**^	7.69 ± 18.29 Median, range: 2 (0-68)	5.30 ± 9.85 Median, range: 4 (0-52)	0.598
**Macular center**	244.53 ± 25.46	250.61 ± 21.58	0.440
**Primacula superior**	325.23 ± 28.78	331.84 ± 22.47	0.435
**Primacula inferior**	324.30 ± 29.03	328.84 ± 22.06	0.589
**Primacula temporal**	313.00 ± 24.32	315.84 ± 17.95	0.681
**Primacula nasal**	327.23 ± 26.42	328.96 ± 21.84	0.829

The Student t-test was used for all comparisons between the two groups, except for global asymmetry and hemisphere S-I which Mann-Whitney test was used.

^a^*DM* Diabetes mellitus

^b^The absolute difference of RNFL thickness between superior and inferior hemisphere

## Discussion

We revealed that the CMT, as well as macular thickness in all quadrants, were significantly thinner in TDT patients compared to healthy individuals. However, RNFL measurements were comparable between the two groups.

Abnormal RNFL changes have been previously reported in certain ophthalmologic diseases, mainly glaucoma [12] and with less frequency in axial myopia [13]. Moreover, it is observed in some systemic diseases, like multiple sclerosis, Alzheimer’s disease, Parkinson’s disease [14], and familial hypercholesterolemia [15].

Chronic anemia, tissue hypoxia, iron overload, iron-mediated oxidative stress, and ICT toxicity are possible contributing factors of the retinal abnormalities in patients with TDT [1, 16].

Although iron is an essential element in several cellular reactions in neural tissue; excessive iron may be toxic due to the releasing of reactive oxygen species leading to destructive changes in the retina and optic nerve [17]. In this context, significant thinner average RNFL, as well as thinner RNFL almost in all quadrants, were reported in children with β-thalassemia major (β-TM) compared to healthy individuals possibly due to iron excess and ICT toxic effects in Aksoy et al [18] and Uzun et al [7] studies. In contrast, in our study, none of the RNFL thickness measurements showed a significant difference between patients and healthy individuals. Furthermore, we did not find any significant correlation between RNFL thicknesses with age, serum ferritin, or hemoglobin levels almost in all measurements except for a significant negative correlation between superior RNFL thickness and serum ferritin level. However, based on the small number of patients and relatively low power of this relationship in the present study, the results should be interpreted by caution and further assessment with larger sample size is recommended in future studies. Likewise, Ulusoy et al [6] reported no significant difference between RNFL thickness in patients with β-TM and healthy controls. They found no significant correlation of RNFL thickness with serum ferritin or hemoglobin level. Similar results were reported in patients with β-thalassemia minor compared to healthy individuals [16, 19]. Besides, Uzun et al [7] showed no significant association between RNFL thickness and age, hemoglobin, or serum ferritin level. Contrary to our results, Aksoy et al [18] showed a significant positive correlation of RNFL thickness with hemoglobin and a significant negative correlation with ferritin. It should be noticed that patients both in Ulusoy et al [6] study and in our study were older than Aksoy et al [18] and Uzon et al [7] studies. As we know, children with β-TM have shorter axial length due to significant retardation in ocular growth [20], and maybe it is more prominent in childhood and improves with age. On the other hand, RNFL thickness and optic nerve topography are likely affected by age, race, and axial length [9, 21–23]. So, some of these discrepancies can be related to the difference in age, race, and axial length in different study populations.

Based on our results, all macular thickness measurements were significantly thinner in patients with TDT compared to controls. Similarly, El-Shazly et al [24] detected a significant thinner foveal thickness in children with β-TM compared to controls, and it was more noticeable in patients who were taking DFO in comparison with deferasirox. The latter finding was inconsistent with our study, as we found no significant association of macular thickness measurements with ICT type. Besides, Arifoglu et al [19] showed a significant thinner macular thickness in all quadrants in adult patients with β-thalassemia minor compared to healthy individuals. It seems that chronic anemia can be a contributing factor in macular thinning.

On the other hand, we found a significantly negative correlation with high power between hemoglobin and CMT as well as significantly negative correlations with low power between hemoglobin and temporal and nasal macular thickness in patients with TDT. In line with this result, anemia has been recognized as a significant risk factor in developing macular edema in diabetic patients [25–27]. So, we hypothesize that the severity of anemia in TDT patients can cause macular swelling in some extent. However, documenting this issue in thalassemia patients needs further evaluations with larger sample size. In contrast to our results, Ulusoy et al [6] found no significant difference in the CMT of patients with β-TM and healthy individuals.

Several lines of evidence showed that ocular toxicity of ICT mainly with DFO resulting in RPE changes with a higher risk for intravenous DFO compared to subcutaneous consumption [3, 28, 29]. Although we found no significant association between RNFL or macular thickness measurements and types of ICT regimen, the role of the iron-chelating agents on the thickness and the function of the optic nerve is not well known and needs further investigations [7]. In our study, approximately 70% of the patients were taking DFO as monotherapy or combination therapy with DFX/ DFP. Moreover, in the DFX group, the patients had been previously on DFO therapy, so we were not able to accurately evaluate the effect of the ICT regimen on RNFL and macular thickness.

In the present study, abnormal findings via posterior segment examinations were observed in three patients including one large CD ratio (2.6%), one vessel tortuosity (2.6%), and one RPE mottling (2.6%). Similarly, Aksoy et al [18] reported a small number of abnormal fundus examinations, including RPE mottling in three patients, and retinal pallor in one patient from a total of 47 children with β-TM. However, a higher frequency of RPE mottling (25%) and increased CD ratio (37.7%), as well as 12.5% disk hyperemia, were detected in children (6-16 years) with β-TM by Abdel-Malak et al [4] They stated that these involvements were observed to be more prevalent in the younger age. Therefore, the fewer fundus abnormalities in our patients might be related to the older age range in our study. Additionally, Taneja et al [1] showed a frequency of 31% RPE degeneration, 9% RPE mottling, 11% venous tortuosities, 7% disc hyperemia, and 4% increased cup-disc ratio in patients with β-TM between six months and 21 years of age.

Based on our results, the most frequent findings in OCT (multicolor fundus photo and BAF) of both eyes consisted of tigeroid patches (circumscribed areas of retinal pigment epithelial thinning) [30]: 13 (16.6%), granularity: 10 (12.8%), foveolar or peripheral darkening 8 (10.2%), hypo autofluorescence patch 6 (7.7%) and preretinal vitreous cell and condensations: 6 (7.7%). Notably, pseudoxanthoma elasticum (PXE)-like retinal abnormality was determined only in one patient as optic disk drusen in a 21-year-old non-diabetic male with hemoglobin: 10 g/dl and ferritin 1400 ng/ml. This abnormality had not been determined in fundus examination and discovered by fundus image.

Granuloid-like accumulations at the retinal pigment epithelium level presenting as black sunburst lesions detected by OCT was previously reported in a 38-year-old man with β-thalassemia intermedia associated with angioid streaks. Granule penetration was observed in the inner layers of the macula and choroid as well as at the photoreceptor layer [31]. In our study, granularity was noticed in 12.8% of the eyes in TDT patients mainly located in the temporal quadrant of the macula, posterior pole, and at the photoreceptor layer.

RPE hypertrophy as enlargement of the RPE cells projected into the subretinal space has been previously reported in the postmortem eye from a patient with β-TM. Moreover, RPE cell structural variations, swollen mitochondria, thickened Bruch membrane, and loss of basal plasma membrane infolding was detected by transmission electron microscopy possibly as a result of iron overload and DFO toxicity [32]. In our study, preretinal vitreous cells and condensations occurred in 7.7% of the eyes.

Based on the results of a meta-analysis on five cross-sectional studies, the prevalence of RPE abnormalities in β-TM patients was reported as 29% CI: 22%-36%, angioid streak (0.01 (0-0.03) and retinal vessel tortuosity 0.08 (0.02-0.15) [33]. The association of increased vascular tortuosity with chronic anaemia was shown in patients with β-TM due to tissue hypoxia, especially in elderly patients, and significant reduction of the hematocrit. Moreover, it was reported in correlation with higher Aspartate Aminotransferase and ferritin levels as well as a higher rate of splenectomy [5]. likely due to hypercoagulable state induced by splenectomy [34]. Retinal vasculature can be directly visualized and may be affected in many conditions such as systemic hypertension, diabetes mellitus, or hypoxia [16]. It is noteworthy that vessel tortuosity in our study was observed in a 43-year-old splenectomized diabetic female (DM duration: 19 years), hemoglobin: 9.8 g/dl and ferritin 2456 ng/ml, having the identified risk factors including older age, DM, splenectomy, and high ferritin level.

Comparison of the measurements between TDT patients with and without DM, showed non-significant thinner values for all macular thickness measurements in TDT patients with DM compared to those values in TDT patients without DM. It might be significant with a larger number of patients. However, the macular thickness was not significantly correlated with DM duration. In contrast, Asefzadeh et al [35] reported a significant correlation between macular and foveal thickness and duration of disease, reflecting neurodegenerative changes in the diabetic retina. Similar to our results, they showed a non-significant difference in macular thickness in diabetic patients compared to healthy individuals.

Our study was limited due to small sample size in subgroup analysis of thalassemia patients. However, we performed power analysis for all significant findings and discussed in details. Also, we did not evaluate the axial length of the patients which can affect the retinal measurements. Moreover, we were not able to assess the effect of different kinds of iron chelation agents on the retina separately, because more than half of our patients have been used a combined regimen of two iron chelation agents. Also, before our study, they might use other kinds of ICT influencing the results.

In conclusion: Macular thickness was significantly thinner in the central macula as well as entire quadrants in TDT patients in comparison with healthy individuals. The significant negative correlation of hemoglobin with central macula thickness detected in thalassemia patients suggests the possibility of macular swelling due to severity of anemia. All RNFL measurement thicknesses were comparable between the patients and controls. Macular thickness was insignificantly thinner in patients with DM compared to non-DM. Close monitoring of TDT patients by periodic ophthalmologic examinations with more focus on diabetic patients, patients with severe anemia and iron overload should be warranted. Further larger multicenter studies are suggested to more accurately evaluate structural ocular changes and associated factors in thalassemia patients.

## Data Availability

The datasets used and/or analysed during the current study available from the corresponding author on reasonable request.

## Abbreviations

TDT: Transfusion-dependent β-thalassemia
OCT: Optical coherence tomography
RNFL: Retinal nerve fiber layer
DM: Diabetes mellitus
ICT: Iron chelation therapy
IOP: Intraocular pressure
BAF: Blue light autofluorescence
SD-OCT: Spectral Domain Optical Coherence Tomography
ETDRS: Early Treatment Diabetic Retinopathy Study
SD: Standard deviation
DFO: Deferoxamine
DFP: Deferiprone
DFX: Deferasirox
CD: Cup-to-disk
RPE: Retinal pigment epithelium
CMT: Central macular thickness
β-TM: β-thalassemia major
PXE: Pseudoxanthoma elasticum
